# Detection and characterization of *Brucella* spp. in bovine milk in small-scale urban and peri-urban farming in Tajikistan

**DOI:** 10.1371/journal.pntd.0005367

**Published:** 2017-03-15

**Authors:** Elisabeth Lindahl-Rajala, Tove Hoffman, David Fretin, Jacques Godfroid, Nosirjon Sattorov, Sofia Boqvist, Åke Lundkvist, Ulf Magnusson

**Affiliations:** 1 Department of Clinical Sciences, Division of Reproduction, Swedish University of Agricultural Sciences, Uppsala, Sweden; 2 Department of Medical Biochemistry and Microbiology, Zoonosis Science Center and Department of Medical Science, Uppsala University, Uppsala, Sweden; 3 Unit Bacterial Zoonoses of livestock, Operational Direction Bacterial Diseases, Veterinary and Agrochemical Research Center, Brussels, Belgium; 4 Faculty of Biosciences, Fisheries and Economics, Department of Arctic and Marine Biology, University of Tromsø - the Arctic University of Norway, Tromsø, Norway; 5 Center for National Collection of Pathogenic Microorganisms, Institute of Biosafety Problems, Tajik Academy of Agricultural Sciences, Dushanbe, Tajikistan; 6 Department of Biomedical Sciences and Veterinary Public Health, Division of Food Safety and Bacteriology, Swedish University of Agricultural Sciences, Uppsala, Sweden; Fondation Raoul Follereau, FRANCE

## Abstract

Brucellosis is one of the most common zoonoses globally, and Central Asia remains a *Brucella* hotspot. The World Health Organization classifies brucellosis as a neglected zoonotic disease that is rarely in the spotlight for research and mainly affects poor, marginalized people. Urban and peri-urban farming is a common practice in many low-income countries, and it increases the incomes of families that are often restrained by limited economic resources. However, there is a concern that the growing number of people and livestock living close together in these areas will increase the transmission of zoonotic pathogens such as *Brucella*. This study investigates the presence of *Brucella* DNA in bovine milk in the urban and peri-urban area of Dushanbe, Tajikistan. *Brucella* DNA was detected in 10.3% of 564 cow milk samples by IS*711*-based real-time PCR. This finding is concerning because consumption of unpasteurized dairy products is common in the region. Furthermore, *Brucella* DNA was detected in the milk of all seropositive cows, but 8.3% of the seronegative cows also showed the presence of *Brucella* DNA. In addition, sequence analysis of the *rpoB* gene suggests that one cow was infected with *B*. *abortus* and another cow was most likely infected with *B*. *melitensis*. The discrepancies between the serology and real-time PCR results highlight the need to further investigate whether there is a need for implementing complementary diagnostic strategies to detect false serological negative individuals in *Brucella* surveillance, control, and eradication programmes. Furthermore, vaccination of cattle with S19 in addition to vaccination of small ruminants with Rev 1 might be needed in order to control *Brucella* infections in the livestock population but further research focusing on the isolation of *Brucella* is required to obtain a comprehensive understanding of the *Brucella* spp. circulating among the livestock in this region.

## Author summary

Central Asia continues to be a hotspot for brucellosis among humans and livestock. The WHO classifies brucellosis as a neglected zoonosis that is rarely in the spotlight for research and mainly affects poor, marginalized people. One of the most powerful megatrends of our time is urbanization, and an often forgotten consequence of human urbanization is the urbanization of their livestock. When large concentrations of humans and livestock build up in urban areas, there is a concern that the transmission of zoonoses like brucellosis will increase. Our results indicate that *Brucella* DNA is widespread in dairy milk in the urban and peri-urban area of Dushanbe, the capital of Tajikistan. This could constitute a public health risk because consumption of raw dairy products is common in the region. Furthermore, a discrepancy between the results of serology and PCR suggests that implementing complementary diagnostic strategies to detect false serological negative individuals might be warranted in *Brucella* control programmes.

## Introduction

Brucellosis is considered to be one of the most common zoonotic infections worldwide with major public health implications [1], but it is still classified as one of seven neglected zoonotic diseases by the World Health Organization (WHO) [2]. The global incidence of human brucellosis is estimated to exceed 800,000 cases per year, of which 40% are estimated to result in a chronic infection [3]. Central Asia and the Middle East are areas with high incidence rates among humans and livestock. Deregulations of trade and decreased border controls following political changes in post-communist countries are believed to be one set of explanations as to why Central Asia is currently a hotspot for brucellosis [4]. One of the most powerful megatrends of our time, in Asia as well as globally, is urbanization [5], but an often forgotten consequence of human urbanization is the urbanization of their livestock [6]. Urban and peri-urban (UPU) livestock production contributes to the supply of fresh food and income for families that are often restrained by limited economic resources [7]. However, there is a concern that the growing number of people and livestock living close together in UPU areas will increase the transmission of different zoonotic pathogens such as *Brucella* [6]. Small-scale UPU farming is a common practice in many low-income countries and in Tajikistan approximately 80% of the population is represented by small-scale livestock farmers [8].

There are currently 12 different species described within the genus *Brucella* [9, 10]. The species mainly concerning livestock and their principal farm animal hosts are *Brucella abortus* (cattle), *B*. *melitensis* (sheep and goats), and *B*. *suis* (swine), and all have a zoonotic potential except for *B*. *suis* biovar 2 [11]. Disease transmission to humans most commonly occurs after direct contact with an infected animal or through consumption of unpasteurized dairy products [12]. If acute human brucellosis is not treated with adequate antibiotics, the infection can turn into a chronic disease and lead to permanent disability [13]. The disease in livestock mainly affects the reproductive organs and the udder and retromammary lymph nodes are often permanently infected in cows [12]. Frequent shedding of *Brucella* into the milk constitutes a risk for the consumers of unpasteurized dairy products.

Serology is widely used in surveillance and control programmes for brucellosis, but serological assays can give false-positive [14], or false-negative [15–17], results. Furthermore, serology tests do not reveal which *Brucella* spp. is causing infection in the host, and this precludes the possibility of identifying the infection source which is important to know when planning and implementing appropriate control measures [18].

Bio-safety level 3 laboratories are recommended for cultural growth of all zoonotic *Brucella* spp. that infect livestock due to the very low infectious dose [19–20]. In many low-income countries, there are few or no bio-safety level 3 laboratories available. Genetic characterization using molecular DNA technology allows molecular typing of *Brucella* without having to handle living *Brucella* organisms [21]. The quantitative or real-time polymerase chain reaction (qPCR) assay targeting the insertion element IS*711* is specific and highly sensitive and could be an appropriate method for the rapid and safe detection of the genus *Brucella* [22]. Further classification of *Brucella* at the species level can be performed by qPCR targeting the *rpoB* gene [23].

The objectives of the current study in the UPU area of Dushanbe, Tajikistan, were to investigate the presence of *Brucella* DNA in bovine milk with qPCR, to perform sequence analysis of *Brucella* DNA extracted from bovine milk, and to investigate how the qPCR result corresponds to previously obtained serology data.

## Materials and methods

### Ethics statement

Samples were collected in compliance with EU legislation on research involving animals [24], and the animals were treated according to the ethical standards of Tajik Agrarian University. The study protocol that included non-invasive collection of milk samples by traditional hand-stripping was approved by the “Ethic committee of the Tajik Agrarian University” (Dushanbe, Tajikistan). The farmers were informed about the purpose and methods of the study and that participation was on a voluntary basis. Informed verbal consent was obtained from all participants and documented together with the coordinates of each herd. All data was handled anonymously and no data regarding the identity of individual animals or farmers were collected. This set-up was important because the farmers would not receive any financial compensation if a cow was found to be *Brucella* spp.-positive and thus at risk of being culled. Therefore, collecting personal data would risk many farmers to refuse to participate in the study.

### Study area and study population

**Fig 1:**
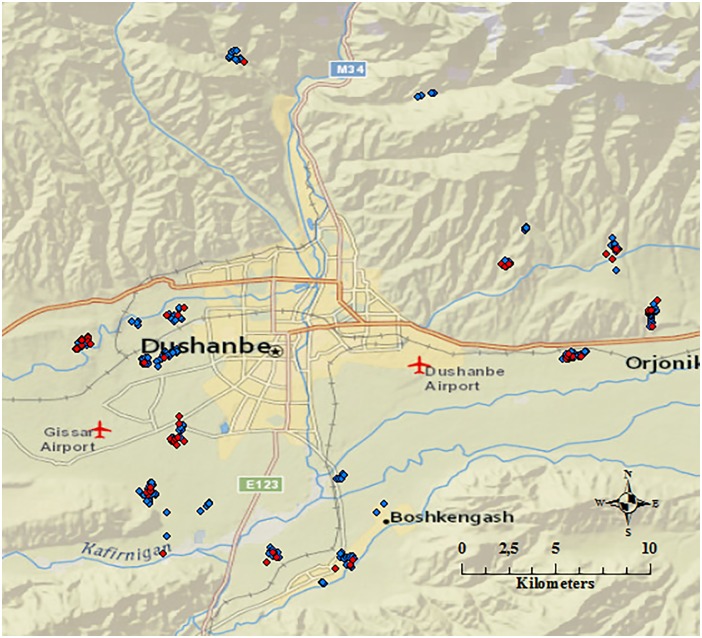
Map of the study area and results from IS*711*-based qPCR at herd level (n = 324). Positive herds (n = 52) are represented by red dots and negative herds (n = 272) are represented by blue dots (ArcGIS software by Esri, www.esri.com).

The study area and study population have been described in detail previously [25]. In brief, the current study was conducted in the UPU areas of Dushanbe, the capital city of Tajikistan, with a radius of <20 km from the central part of the city (Fig 1). There are approximately 800,000 people living in Dushanbe [26] and the UPU area is dominated by small-scale farming with approximately 45,000 dairy cows in the study area. The villages within the UPU areas practice either communal grazing on natural rangelands or keep their animals tethered or at limited pastures. Rearing sheep and goats together with cattle is common practice in the peri-urban areas where the villages have access to natural rangelands. The predominant dairy cow is a local breed with an estimated average annual milk production of 3,000 liters.

### Study design and collection of individual milk samples

This study was carried out simultaneously with a seroprevalence study among dairy cows where the selection of villages, herds and individuals has been described previously in detail [25]. In brief, information of the villages keeping dairy cows with a radius of < 20 km from the central part of Dushanbe was received from the local official veterinarians and the villages were numbered and selected randomly. In each village, as many herds as possible with dairy cows were sampled. In each herd the aim was to sample all lactating cows. The seroprevalence study included 904 cows in 443 herds, and among these milk samples were collected from 570 cows in 329 herds. Thus the current report comprise 570 cows with serological data. In the current study, approximately 2 mL of milk was collected from each cow into Eppendorf tubes and kept cold during transport to the laboratory at the Tajik Agrarian University in Dushanbe. In the seroprevalence study, serum samples were tested for *Brucella*-specific antibodies with indirect enzyme-linked-immunosorbent assay (I-ELISA), and positive samples were confirmed with competitive ELISA (C-ELISA). None of the cattle in the study had been vaccinated against brucellosis according to information from the local official veterinarians. A GPS (Global Positioning System) receiver was used to record coordinates (latitude/longitude) of the herds. The milk was inactivated at 56°C for 30 min and then stored at -20°C until transport to the Zoonosis Science Center at Uppsala University, Sweden.

### DNA extraction from milk samples

The molecular analyses were performed at the Zoonosis Science Center at Uppsala University, Sweden, and at the Veterinary and Agrochemical Research Center in Brussels, Belgium. Bacterial DNA was extracted from the milk samples using phenol:chloroform:isoamyl alcohol (Sigma-Aldrich, Saint Louis, Missouri, US) according to a protocol for extraction of bacterial DNA from milk and cream recommended by the *Brucella* reference laboratory at the Animal Health and Veterinary Laboratories Agency (AHVLA) (Weybridge, UK). Milk samples were randomly chosen for extraction in sets of 24 to avoid cross-contamination between samples and the DNA extracts were stored at -20°C. The milk extracts were analysed for inhibition and presence of bacterial DNA according to a protocol by Corless et al. [27] with universal primers and probe (Thermo Fisher Scientific, Massachusetts, US) targeting the bacterial *16S*-gene, with the minor modification of reducing the template volume to 1 μL. Samples were evaluated as positive when the cycle threshold (Ct) value was less than the negative control. The baseline was set using the normalization method: dynamic tube normalization (default setting/auto-baseline) in the Rotor-Gene software 2.1.0.9, while the threshold was set manually at 0.020. The expected amplicon length was 111 base pairs (bp). DNA from the bacterial strain T2378 of *Treponema* sp. and *Pseudomonas aeruginosa* strain B683, were used as positive controls in the *16S* rRNA qPCR assay, and sterile water was used as negative control.

### IS*711* qPCR

The *Brucella* genus-specific insertion element IS*711* was targeted during screening of *Brucella* spp. The primer-probe set came from Matero et al. [28]. In brief, the IS*711* amplification reactions contained: 2 μL DNA template, 2.5 U AmpliTaq Gold DNA polymerase (Applied Biosystems, Foster City, California, US), 1X (5 μL of 10X) GeneAmp buffer II, 6 mM MgCl_2_, 800 μM GeneAmp dNTP blend (Applied Biosystems, California, United States), 300 nM of each primer (Thermo Fisher Scientific, Massachusetts, US), 250 nM probe (Life Technologies, Carlsbad, California, US), and sterile water. The final reaction volume was 50 μL, and the amplification profile was as follows: a hot-start step at 95°C for 10 min followed by 45 cycles of 95°C for 15 s and 60°C for 60 s. The expected amplicon length was 53 bp. A sample was considered to be positive if the cycle threshold (Ct) ≤ 38. All samples were analysed twice and a sample was considered to be *Brucella* spp.-positive if the qPCR showed positive test results in both runs. The baseline was set using the auto-baseline (normalization method: dynamic tube normalization) in the Rotor-Gene software 2.1.0.9, while the threshold for the IS*711*-assay was set manually at 0.055. In all assays, two positive controls consisting of DNA of the reference strain *B*. *suis* biovar 1 from the commercial INgene Bruce-ladder V kit, Ingenasa, Madrid, Spain were included. A negative control containing sterile water and a no-template control were included in all qPCR runs. Additionally, an internal inhibition/amplification control containing master mix, one part randomly chosen extract and one part positive control was included in each run. Inhibition of the IS*711* assay in the cow milk matrix was analysed by diluting extracts that gave no signal in the *16S* assay, got Ct-values higher than the negative control in the *16S* assay, and/or had a Ct-value around 40 in the IS*711* assay, 10 and 100 fold in sterile water. Amplification and fluorescence measurements were performed on a Rotor-Gene 6000 qPCR machine (Corbett Research, Mortlake, Australia). The efficiency and the sensitivity of the IS*711* qPCR assay were evaluated using two 10 fold serial dilutions of the positive control, prepared in sterile water and a PCR-negative pool of milk extracts. The DNA concentrations, decided by NanoDrop 2000c Spectrophotometer (Thermo Scientific, Wilmington, Delaware, USA), tested were: 2.90 ng μL-1, 290 pg μL-1, 29.0 pg μL-1, 2.90 pg μL-1, and 290 fg μL-1.

### DNA sequencing

It has been suggested that it would be possible to identify isolates at the species and possibly biovar level by sequencing the *rpoB* gene of an unidentified *Brucella* isolate/DNA and querying a database such as GenBank [23]. Single nucleotide polymorphisms (SNPs) have been documented at 22 positions in the 4093 bp sequences of classical *Brucella* species. A tentative identification of the *Brucella* species present in milk samples was performed on the extracted DNA. We designed 2 sets of primers (Primer design tool Primer-BLAST, https://www.ncbi.nlm.nih.gov/tools/primer-blast/) to amplifying regions including SNP 716, 737, 969 and 985.

Set of primers 1: rpoB1983F: AAGCAGCTTGTTTCGGTTGC/rpoB2193R: GACCTGATCGACGATACCG

Set of primers 2: rpoB2722F: TTCGGTGAAAAGGCATCCGA/rpoB3119R: AGCAGCTTCTTGGAGTCGTC

The first set of primers (rpob1983F/rpob2193R) allows the amplification of a fragment of 210 bp that includes SNPs at positions 716 and 737 while the second set of primers (rpoB2722F/ rpoB3119R) allows the amplification of a fragment of 397 bp that includes SNPs at positions 969 and 985. PCR amplifications were carried out using Icylcer Bio-rad PCR System (Bio-rad, Temse, Belgium), following the Taq Polymerase manufacturer’s suggested protocol (TermoFisher, Gent, Belgium) for reaction. Amplifications were initiated by denaturing the sample for 2 min at 94°C, followed by 40 cycles at 94°C for 45 s, 55°C for 30 s, and 72°C for 45 s. After the last cycle, samples were incubated for an additional 10 min at 72°C before they were stored at 4°C. Ten microlitres of each reaction mixture were analysed by electrophoresis through a 1% agarose gel with ethidium bromide. Custom DNA sequencing was performed by Macrogen DNA Sequencing Service, Amsterdam, the Netherlands. Sequences were pairwise aligned and compared to the previously determined *rpoB* sequence of *B*. *abortus* strain 2308 using programs provided by the National Center for Biotechnology Information (www.ncbi.nlm.nih.gov).

### Accession numbers

*B*. *abortus* strain 2308 accession number AY562179

## Results

### Detection of *Brucella* DNA with IS*711* qPCR and corresponding serology results

**Fig 2:**
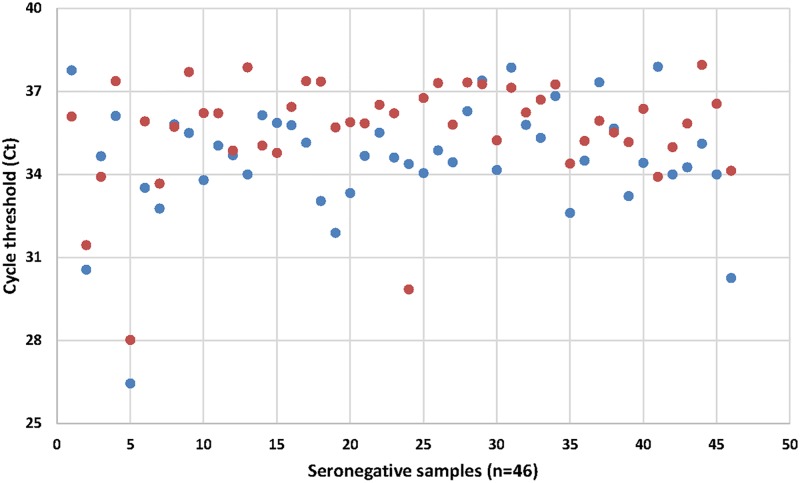
Ct-values from the IS*711*-based qPCR of the seronegative individuals (n = 46). The blue dots represent Ct-values from the first run and the red dots represent Ct-values from the second run.

In total, 570 cow milk samples were collected. DNA could not be extracted from two samples, resulting in 568 DNA extracts. Four additional milk samples were excluded from the study due to low amounts of extract. Consequently, 564 cow milk extracts from 326 herds in 21 villages were analysed for the presence of *Brucella* DNA. GPS coordinates were recorded for all but two herds (n = 324) (Fig 1) (Arc Map 10.4.1, ArcGIS software by Esri, www.esri.com). In total, bacterial DNA was present in 88% (n = 486) of the extracts analysed with the *16S* assay. Twelve samples had to be excluded from the *16S* assay due to low amount of extract. *Brucella* DNA was detected in 10.3% (n = 58) of the milk samples with IS*711* qPCR. The internal inhibition/amplification control was positive in each assay. The apparent individual seroprevalence measured previously with I-ELISA and C-ELISA was 2.1% [25]. All seropositive cows (n = 12) were also positive in the IS*711* qPCR with Ct-values ranging between 26.9 and 31.9. Out of the 552 seronegative cows, 8.3% (n = 46) were *Brucella* positive by IS*711* qPCR with Ct-values ranging between 26.5 and 38.0 (Fig 2). At herd level, 16% (n = 52) of the herds had at least one positive cow based on IS*711* qPCR (Fig 1). In total, 14.9% (n = 84) of the extracts showed signs of inhibition in the *16S* or in the IS*711* assay. After dilution, only nine DNA extracts proved to contain PCR inhibitors affecting the IS*711* qPCR assay. The efficiency of the IS*711* qPCR assay was >99% (R2 = 0.999) in sterile water, 91% (R2 = 0.996) in undiluted cow milk matrix, and 96% (R2 = 0.999) in 10 fold diluted milk matrix. The IS*711* qPCR detected the lowest concentration of *B*. *suis* DNA tested in this study, 290 fg μL-1 in water, undiluted milk, and diluted milk.

### *Brucella* DNA analysis

Fourteen samples, with Ct-values ranging between 26.5 and 30.6, were selected for further analysis to species level but only two samples had sufficient amounts of DNA to perform sequence analysis. The first sample was collected from a seropositive cow, and the SNP allelic profiles corresponded to the profiles described for *B*. *melitensis* and *B*. *suis* at codon positions 716 and 737 [23] (Table 1) (S1 File). The other sample came from a seronegative cow, and the SNP allelic profiles corresponded to *B*. *abortus* at codon positions 716, 969, and 985. At codon position 737, which has previously been reported to be GTC for *B*. *abortus* (23), the SNP was not described for *B*. *abortus*. See Table 1 for accession numbers of sequences deposited in GenBank (www.ncbi.nlm.nih.gov/genbank/).

**Table 1:** Sequence analysis of the *RpoB*-gene.

Sample	Codon position				
	716	737	969	985	
1	CCG^1^	GTT^1^			Data in S1 File
2	CCA^2^	GTT	CGT^2^	GCC^2^	Accession numbers: KY678717 & KY678718

^1^Corresponds to *B*. *melitensis* and *B*. *suis* [23].

^2^Corresponds to *B*. *abortus* [23].

## Discussion

This study shows that *Brucella* DNA was commonly detected in bovine milk in the UPU area of Dushanbe, both among *Brucella* seropositive and seronegative individuals. DNA sequence analysis suggests that one cow was infected with *B*. *abortus* and that another cow most likely was infected with *B*. *melitensis*.

In total, 10.3% of the cows had *Brucella* DNA in their milk as measured by IS*711*-based qPCR. The corresponding figures among the seropositive and seronegative cows were 100% and 8.3%, respectively. A similar discrepancy between the serology and qPCR results was demonstrated in a study from Switzerland comparing IS*711*-based qPCR, serology, and culture among wild boars in which *Brucella* DNA was detected in tissue samples of 11.1% of the seronegative individuals [29]. The discrepancy between the serology and qPCR results observed in the current study might indicate that the true number of *Brucella*-infected cattle within the study area is underestimated by serology screening. Serological false negative results have been reported as a consequence of reduced antibody titers over time. Hence, seronegative animals in the current study that tested positive by IS*711*-based qPCR might have been previously exposed to *Brucella* and then turned seronegative after a certain time period [30]. Another factor that might influence the result is sampling at an early stage of the infection, i.e. within the first 14 days, when the humoral immune response has not yet produced detectable levels of antibodies in the host [31]. Furthermore, individuals infected in utero or in the early post-natal period can become latently infected and thus never become serologically positive [12], and approximately 3.5% of infected cows are estimated to deliver latent infected offspring [32]. It has also previously been reported that *B*. *suis* infection in cattle generates a shorter duration of antibody response in the host [15]. Whether this is also true for *B*. *melitensis* infection in cattle is not yet known and needs to be investigated further. If this is the case, it might partially explain the discrepancy between the serology and qPCR results observed in the current study. Another plausible explanation for the discrepancy between the serology and qPCR results might be previous vaccination against brucellosis [33]. However, in this study, the information given from the local official veterinarians that none of the cattle had been vaccinated against brucellosis is considered reliable because there is no national control programme for brucellosis among livestock in Tajikistan.

The results from the sequence analysis of the *rpoB* gene suggests that *B*. *abortus* was present in the milk of one dairy cow. The analysis also revealed an SNP for *B*. *abortus* that has not previously been described, but whether this SNP is a new marker for *B*. *abortus* in the region remains unclear and more research is required to draw firm conclusions. Analysis of the other sample showed SNPs compatible with both *B*. *melitensis* and *B*. *suis*; however, because pig production is almost non-existent in Tajikistan, it is highly likely that this cow was infected with *B*. *melitensis*. This cow was not being kept together with small ruminants at the time of sampling, and the source of infection in this particular case remains unknown. The prevailing epidemiological situation in the study area, with endemic *B*. *melitensis* infection among sheep and goats [34] and where cattle are often kept in close proximity with small ruminants, suggests a spillover of *B*. *melitensis* from small ruminants to cattle which has also been demonstrated in a study from the neighboring country of Kyrgyzstan where *B*. *melitensis* has been isolated from cattle [35]. In the current study, only two samples yielded a sufficient amount of DNA to perform sequence analysis. Thus further research, including isolation of *Brucella* spp. from cattle, sheep, and goats, is required in order to obtain a comprehensive understanding of the *Brucella* spp. circulating within the livestock population in this region.

Drawing firm conclusions regarding the zoonotic risk of consuming the milk from the qPCR- positive cows is difficult because qPCR can detect DNA from live, damaged or dead bacteria. However, because consumption and trading of unpasteurized dairy products is common among small-scale farmers in the UPU area of Dushanbe [36], the significant numbers of cows with detectable levels of *Brucella* DNA in their milk might constitute a serious health concern.

The IS*711*-based qPCR is very sensitive with a detection limit of 10 copies [37], and potential bias in the current study might have arisen due to DNA contamination, either during sample collection or in the laboratory. During the sample collection, gloves were used as a protective measure and were changed between samplings at each household. The extraction of DNA from milk and the IS*711*-based qPCR was conducted in a laboratory where little work on *Brucella* had been conducted, and thus the risk of *Brucella* contamination within the laboratory was low. With the measures taken, we believe that we have minimized the risk of contamination and consider the results presented in the current study to be representative for the study population.

## Conclusions

This study shows widespread occurrence of *Brucella* DNA in bovine milk in the UPU area of Dushanbe. Furthermore, our results suggest that one cow was infected with *B*. *abortus* and another cow was most likely infected with *B*. *melitensis*. Thus, vaccination of cattle with S19 in addition to vaccination of small ruminants with Rev 1 might be needed in order to control *Brucella* infections in the livestock population but further research focusing on the isolation of *Brucella* is required to obtain a comprehensive understanding of the *Brucella* spp. circulating among the livestock in this region. The discrepancies between the serology and qPCR results, i.e. the potentially significant number of false serological negative individuals in the current study, highlights the need to further investigate whether there is a need for implementing complementary diagnostic strategies to detect false serological negative individuals in *Brucella* surveillance, control, and eradication programmes.

## Supporting information

S1 File Sequence analysis of the *RpoB*-gene (sample 1). (DOCX)
