# Supplementary material for: Detection and characterization of Brucella spp. in bovine milk in small-scale urban and peri-urban farming in Tajikistan
Source: PLoS Negl Trop Dis. 2017 Mar 15;11(3):e0005367. doi: 10.1371/journal.pntd.0005367 (PMC5367834; doi:10.1371/journal.pntd.0005367)
Supplement: S1 File — (DOCX) [file pntd.0005367.s001.docx]

gaacatgcagcgtcaggctgtgccgctcgtgcgtgctgaagcgccgtttgtcggcacgggcatggaa**ccg**atcgtggctcgcgactctggtgcagccattgcagcgcgccgcggcggtatcgtcgatcag**gtt**gat
